# BAK-up: the receptor kinase BAK-TO-LIFE 2 enhances immunity when BAK1 is lacking

**DOI:** 10.1007/s44154-023-00124-y

**Published:** 2023-09-25

**Authors:** Vahid Fallahzadeh-Mamaghami, Hannah Weber, Birgit Kemmerling

**Affiliations:** ZMBP, University Tübingen, Auf der Morgenstelle 32, Tübingen, 72076 Germany

**Keywords:** BAK1, Cell death, Receptor kinase, BTL2, PTI, ETI

## Abstract

BRI1-ASSOCIATED KINASE 1 (BAK1/SERK3) and its closest homolog BAK1-LIKE 1 (BKK1/SERK4) are leucine-rich repeat receptor kinases (LRR-RKs) belonging to the SOMATIC EMBRYOGENESIS RECEPTOR KINASE (SERK) family. They act as co-receptors of various other LRR-RKs and participate in multiple signaling events by complexing and transphosphorylating ligand-binding receptors. Initially identified as the brassinosteroid receptor BRASSINOSTEROID INSENSITIVE 1 (BRI1) co-receptor, BAK1 also functions in plant immunity by interacting with pattern recognition receptors. Mutations in BAK1 and BKK1 cause severely stunted growth and cell death, characterized as autoimmune cell death. Several factors play a role in this type of cell death, including RKs and components of effector-triggered immunity (ETI) signaling pathways, glycosylation factors, ER quality control components, nuclear trafficking components, ion channels, and Nod-like receptors (NLRs). The Shan lab has recently discovered a novel RK BAK-TO-LIFE 2 (BTL2) that interacts with BAK1 and triggers cell death in the absence of BAK1 and BKK1. This RK compensates for the loss of BAK1-mediated pattern-triggered immunity (PTI) by activating phytocytokine-mediated immune and cell death responses.

## Main text

**Fig. 1:**
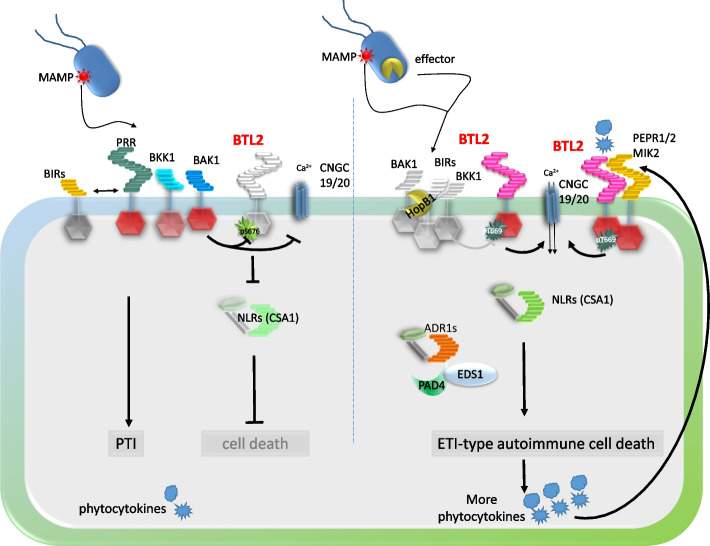
Model of BTL2 function in autoimmune signaling in the absence of BAK1/BKK1 PRR co-receptors. In the absence of BAK1/BKK1, severe autoimmune cell death is activated. The receptor kinase BAK-TO-LIFE 2 (BTL2) can interact with BAK1 and is required for autoimmune cell death in the absence of BAK1 and BKK1. When MAMPs are detected, BAK1/BKK1 dissociate from BIR proteins and are recruited to pattern recognition receptors, activating PTI responses. BTL2 does not contribute to this early process. In the presence of BAK1, BTL2 can be transphosphorylated on residue S676, and BTL2 cell death-inducing capacities are suppressed, suggesting that BAK1/BKK1 negatively regulates autoimmune cell death by suppressing activation of BTL2 by transphosphorylation. Upon perturbation of BAK1/BKK1, either by pathogen effectors like HopB1 or genetic inactivation of these co-receptors, PTI is impaired, but this also results in the activation of BTL2. Autophosphorylation at T669 is required for cell death activation and phosphorylation of CNGC20. Activated BTL2 triggers the activation of CNGC20/CNGC19 calcium channels, leading to Ca^2+^ influx and subsequent autoimmune cell death. The autoimmune cell death is mediated by ETI components such as NLRs (CSA1, ADR1s, and potentially others), SA, and the lipase-like proteins EDS1 and PAD4. PTI signaling induces phytocytokines recognized by cell surface receptors, amplifying PTI responses and establishing robust plant immunity. These receptors also rely on BAK1 as a co-receptor. The massive production of phytocytokines in the absence of BAK1/BKK1 can initiate a feedback loop, exacerbating the cell death reaction. BTL2 is required for these secondary effects and contributes to SCOOP and PEP-triggered responses, including the novel SCOOPL1 peptide.

Plant pattern recognition receptors (PRRs) are cell surface localized and get stimulated by microbe-associated molecular patterns (MAMPs). This leads to the activation of various defense mechanisms, including the production of reactive oxygen species (ROS), activation of MAP kinases, and enhanced expression of pathogenesis-related proteins. This ultimately results in pattern-triggered immunity (PTI) (Bender and Zipfel 2023). Additionally, intracellular NOD-like immune receptors (NLR) can detect microbial effector proteins or their actions and trigger strong defense responses, also including ROS burst, defense gene activation, and a hypersensitive cell death reaction (HR) referred to as effector-triggered immunity (ETI) (Ngou et al. 2022). Both immune systems are interconnected and potentiate each other resulting in robust immunity (Ngou et al. 2021; Pruitt et al. 2021; Tian et al. 2021; Yuan et al. 2021) (Fig. 1).

The Arabidopsis proteins BRI1-ASSOCIATED RECEPTOR KINASE 1 (BAK1) and BAK1-LIKE 1 (BKK1) are both receptor kinases (RKs) localized on plant plasma membranes. They have crucial roles in plant immunity, developmental processes, and autoimmune responses (Ma et al. 2016). Both proteins act as co-receptors interacting with various leucine-rich repeat receptor kinases (LRR-RKs) such as the brassinosteroid receptor BRASSINOSTEROID INSENSITIVE 1 (BRI1) (Li et al. 2002; Nam and Li 2002), the flagellin receptor FLAGELLIN-SENSING 2 (FLS2)(Chinchilla et al. 2007) and the receptor-like protein-interacting RK SUPPRESSOR OF BIR1 (SOBIR1) (Liebrand et al. 2013; Albert et al. 2015). These interactions are crucial for activating downstream signaling pathways involved in plant immunity and development.

RKs can also perceive endogenous plant peptides that function as danger signals, such as SERINE-RICH ENDOGENOUS PEPTIDE (SCOOP) peptides by MALE DISCOVERER 1-INTERACTING RECEPTOR-LIKE KINASE 2 (MIK2) (Coleman et al. 2021; Hou et al. 2021; Rhodes et al. 2021) and PLANT ELICITOR PEPTIDE (PEP) by the PEP RECEPTORS 1 and 2 (PEPR1 and PEPR2) (Yamaguchi et al. 2006, 2010; Krol et al. 2010). These peptides function as phytocytokines that amplify defense responses in a non-cell-autonomous manner. Upon activation, they also recruit BAK1 as a co-receptor and positive regulator of phytocytokine responses. Therefore, BAK1 is involved in primary and secondary events under natural infection conditions.

BAK1 and BKK1 negatively regulate autoimmune cell death. The proper balance of BAK1 and BKK1 and their associated partners is crucial in regulating cell death (Belkhadir et al. 2012; Halter et al. 2014; Dominguez-Ferreras et al. 2015; Imkampe et al. 2017). Any alterations in the amount of BAK1 or its partners can impact the activation of cell death, indicating that this is a quantitatively controlled process.

This type of cell death involves key components of ETI, including ENHANCED DISEASE SUSCEPTIBILITY 1 (EDS1), PHYTOALEXIN DEFICIENT 4 (PAD4), and the ACTIVATED DISEASE RESISTANCE 1 (ADR1) helper NLR family (de Oliveira et al. 2016; Du et al. 2016; Yu et al. 2019a; Wu et al. 2020). These components indicate that an ETI-type surveillance system is monitoring BAK1 and BKK1. CONSTITUTIVE SHADE AVOIDANCE 1 (CSA1), a TIR-domain containing NLR (TNL), has recently been found to play a role in cell death in *bak1 bkk1* and *bak1 bir3* mutants (Schulze et al. 2022; Yang et al. 2022). It interacts directly with BIR3 and indirectly with BAK1 to sense their integrity. When BAK1 is disrupted by genetic measures or following MAMP or effector activation, CSA1 triggers cell death, suggesting that CSA1 guards BAK1 and initiates ETI-type cell death in the absence or imbalance of BAK1 complexes. Additional components are necessary for *bak1 bkk1* cell death, such as nuclear transport components, glycosylation factors, and CYCLIC NUCLEOTIDE GATED ION CHANNELs 20 and 19 (CNGC20/19) (de Oliveira et al. 2016; Du et al. 2016; Gao et al. 2017; Yu et al. 2019a; Wu et al. 2020).

Multiple effector proteins, including HopB1, target BAK1 (Gao et al. 2009). During infection, HopB1 cleaves activated BAK1, resulting in reduced PTI-type defense responses (Li et al. 2016). However, overexpression of HopB1 leads to autoimmune cell death, indicating that perturbation of BAK1 during infection can induce ETI-like autoimmune cell death. HopB1-induced autoimmune responses require the NLR CSA1 and the helper NLR ADR1 family proteins (Wu et al. 2020; Schulze et al. 2022).

BAK1 and BKK1 are closely monitored by BAK1-INTERACTING RECEPTOR 1 (BIR1), BIR2, and BIR3 to ensure appropriate immune responses and prevent unwanted PTI activation in the absence of microbial patterns (Gao et al. 2009; Halter et al. 2014; Imkampe et al. 2017). Loss of BIR1 results in strong autoimmune cell death and loss of BIR2 and BIR3 in weaker autoimmune responses, suggesting that a control system containing additional RKs is necessary to keep BAK1 and BKK1 under control.

In Arabidopsis, BTL2 has been recently identified in an elegant virus-induced gene silencing-based screen of BAK1- and BKK1-silenced plants as a suppressor of autoimmune cell death in BAK1/BKK1 deficient plants (Yu et al. 2023). BTL2 is a cell surface-localized, active non-RD kinase, and kinase activity is required for its function. It belongs to the RK subfamily LRR XI, has 20 LRRs, and is therefore considered a large, likely ligand-binding RK in contrast to the short, only five LRR-containing, not ligand-binding SERK and BIR family receptor kinases (Xi et al. 2019). Whether it is indeed a ligand-binding receptor and what it perceives is an interesting question for the future. Mutants in BTL2 can partially suppress the growth defects and autoimmune phenotypes caused by BAK1 BKK1 silencing or knockout, but none of the tested immune responses are altered in *btl2* single mutants. This shows that BTL2 is not involved in primary plant immunity in the presence of BAK1 and BKK1. In their absence, it is required to induce autoimmune cell death. Overexpression of BTL2 leads to cell death in a significant part of the transformants suggesting a quantitative effect of BTL2 on cell death that BAK1 and BKK1 keep under control in their presence. As shown for BAK1 and BIRs, overexpression of BTL2 leads to enhanced cell death, indicating that imbalances in receptor complexes activate autoimmune cell death (Dominguez-Ferreras et al. 2015; Imkampe et al. 2017; Yu et al. 2023). Other types of autoimmune cell death, such as *bir1-* and MAPK/ERK kinase kinase 1 mutant- (*mekk1)* induced phenotypes, are not suppressed in the absence of BTL2, suggesting independent pathways for these also BAK1-related components. Quantitative effects in different alleles of *bak1* can cause different outcomes in cell death suppression, this might be the case here as well.

As an autoactive kinase, BTL2 autophosphorylates on residue T669. *BTL2*^*T669A*^ mutants cannot restore the growth and autoimmune phenotypes in *bak1 bkk1 btl2* triple mutants indicating that autophosphorylation is required for its activity. In addition, BAK1 can phosphorylate BTL2 at residues T657 and S676, and a phosphomimetic mutant S676D cannot induce cell death anymore, suggesting that phosphorylation of this residue leads to a negative regulation of BTL2 supporting the idea that BAK1 (and BKK1) negatively regulate BTL2 in their presence.

BTL2 enhances the cell death-inducing capacity of CNGC20, and BTL2-induced cell death is impaired in *cngc20* mutants showing that BTL2 can activate CNGC20 in the absence of BAK1/BKK1 and that this activation is necessary to induce autoimmune cell death. BAK1 co-expression with BTL2 and CNGC20 reduces the channel activity, indicating that BTL2 activates and BAK1 suppresses the channel activity (likely via BTL2 phosphorylation). Loss-of-function *cngc20* mutants do not show defects in PTI or ETI though a misregulated gain-of-function allele can affect both (Yu et al. 2019b; Zhao et al. 2021). This supports the model that BAK1 keeps BTL2 and the channel activity under control, and BTL2 activates it in the absence of BAK1/BKK1.

Another previously identified suppressor of *bak1 bkk1* cell death, STAUROSPORIN AND TEMPERATURE SENSITIVE 3a (STT3a), necessary for membrane protein glycosylation, is also essential for BTL2 and CNGC20 glycosylation, and BTL2 glycosylation-deficient mutants are not functional. This shows that proper membrane protein glycosylation is critical for the function of BTL2 and CNGC20.

Phytocytokines such as SCOOP or PEP peptides are produced upon PTI activation and amplify immune responses (Yamaguchi et al. 2006, 2010; Krol et al. 2010; Coleman et al. 2021; Hou et al. 2021; Rhodes et al. 2021). A number of these signaling peptides are upregulated in *bak1 bkk1* mutants. The RK MIK2 mediates SCOOP signaling, and BTL2-induced cell death is reduced in *mik2*. SCOOP and PEP-induced defense responses and immune outcome are also impaired in *bak1 bkk1 btl2* mutants suggesting a role of BTL2 in the amplification and/or perception of phytocytokines in the absence of BAK1/BKK1. This includes a novel SCOOP-LIKE 1 (SCOOPL1) with SCOOP peptide characteristics.

As BTL2 has no impact on immunity in the presence of BAK1 and BKK1, BTL2 likely functions in the amplification loop mediated by phytocytokines, leading to enhanced cell death in *bak1* and *bak1 bkk1* mutants. This cell death likely impacts the amplification of primary responses by activating CNGC20. Exploration of natural conditions in which BAK1 and BKK1 are modified or depleted, leading to activation of BTL2 and compensation of impaired PTI by activating ETI-type immune responses, will be a fascinating future question (Yamada et al. 2016; Zhou et al. 2019; Schulze et al. 2022).

Taken together, BTL2 can suppress cell death occurring in the absence of BAK1 and BKK1 and is involved in phytocytokine signaling but not in primary PTI responses in the presence of BAK1 and BKK1. Interesting open questions are i.) which additional components are required; ii.) how are the multiple components participating in autoimmune cell death organized in the plant cells and iii.) how primary and secondary events are sorted and regulated. BTL2 is an essential factor of BAK1 surveillance, and it will be interesting to see iv.) whether it perceives phytocytokines directly, as a co-receptor of multiple DAMP receptors or as a scaffold for receptor complexes. v.) Sorting out primary and secondary effects in natural infections and specificities for PRRs and phytocytokines receptors might solve the jigsaw of BAK1/SERK-related receptor complexes in plant immunity.

## Data Availability

Not applicable.

## Abbreviations

ADR1: ACTIVATED DISEASE RESISTANCE 1
BRI1: BRASSINOSTEROID INSENSITIVE 1
BAK1: BRI1-ASSOCIATED KINASE 1
BKK1: BAK1-LIKE 1
BTL2: BAK-TO-LIFE 2
CNGC20: CYCLIC NUCLEOTIDE GATED ION CHANNEL 20
CSA1: CONSTITUTIVE SHADE AVOIDANCE 1
EDS1: ENHANCED DISEASE SUSCEPTIBILITY 1
ETI: Effector-triggered immunity
HR: Hypersensitive response
LRR-RK: Leucine-rich repeat receptor kinase
MAMP: Microbe-associated molecular patterns
MAPKs: MITOGEN-ACTIVATED PROTEIN KINASES
MEKK1: MAPK/ERK KINASE KINASE 1
MIK2: MALE DISCOVERER 1-INTERACTING RECEPTOR-LIKE KINASE 2
NLR: Nucleotide-binding domain leucine-rich repeat-containing receptor
PAD4: PHYTOALEXIN DEFICIENT 4
PEP: PLANT ELICITOR PEPTIDE
PRRs: Pattern-recognition receptors
PTI: Pattern-triggered immunity
*Pto*: *Pseudomonas syringae* Pv*. tomato*
RKs: Receptor kinases
RLPs: Receptor-like proteins
ROS: Reactive oxygen species
SA: Salicylic acid
SCOOP(L1): SERINE-RICH ENDOGENOUS PEPTIDE (LIKE 1)
SERK: SOMATIC EMBRYOGENESIS-RELATED KINASE
STT3a: STAUROSPORIN AND TEMPERATURE SENSITIVE 3a
SOBIR1: SUPPRESSOR OF BIR 1
TIR: TOLL/INTERLEUKIN-1 RECEPTOR;
TNLs: TIR NLRs
